# Dynamic elastomeric fabric orthoses in neuropathic scoliosis management: an audit of the frequency and characteristics of use

**DOI:** 10.1186/1748-7161-9-S1-O30

**Published:** 2014-12-04

**Authors:** Suzanna Blandford, John Marsden, Martin Matthews, Jenny Freeman

**Affiliations:** 1 Plymouth University, Plymouth, United Kingdom; 2 DM Orthotics, Redruth, United Kingdom

## Background

Scoliosis is a common consequence of neuromuscular diseases with an incidence of 25% in cerebral palsy (CP) [1] and 75-90% in non-ambulant Duchenne muscular dystrophy [2]. Evidence from case studies demonstrates that dynamic elastomeric fabric orthoses (DEFO’s) may provide an effective alternative to rigid bracing [3], which often causes discomfort and hence is associated with non-compliance.

## Aim

We aimed to describe the routine management of paediatric neuropathic scoliosis, more specifically orthotic management; and explore the progression of scoliosis with DEFO use.

## Design

Retrospective audit.

## Method

The physiotherapy notes of children with neurological conditions were audited in five Healthcare Trusts across England. A standardized data collection form was used to gather diagnostic and demographic information and scoliosis characteristics and management.

## Results

180 notes were audited (85 male; mean age 9 years [SD 4y 7mo]). Diagnoses included cerebral palsy (44%), neuromuscular dystrophy (3%), spinal pathology (2%), developmental delay (23%) and others (28%), including Retts syndrome and epilepsy.

Scoliosis (Cobb angle ≥10 degrees) was confirmed in 77 children of whom 45% had been prescribed a DEFO. This was replaced by a rigid orthoses for 4 children; another 4 stopped using the DEFO or any other form of orthoses. DEFO management was far less likely in those with severe scoliosis. Of those with radiographic/ medical records enabling their scoliosis to be categorized according to severity, 28/42 (72%) with mild scoliosis, 5/8 (63%) with moderate scoliosis and 1/8 (13%) with severe scoliosis used a DEFO. A developing scoliosis was seen in 43 children of whom 51% used a DEFO. No scoliosis was observed in 77 children, all of whom wore a DEFO as a preventative measure. In children wearing a DEFO, in whom the Cobb angle was monitored over time, there was a deterioration of >10^o^ in only 1/8.

## Conclusions

DEFO use varied across region and was used in a variety of neuromuscular conditions. It was used more commonly in the management of less severe scoliosis and as a preventative measure. Where serial monitoring was performed, Cobb angle progression was only minimal in those using a DEFO.
